# Functional coupling of chloride–proton exchanger ClC-5 to gastric H^+^,K^+^-ATPase

**DOI:** 10.1242/bio.20136205

**Published:** 2013-11-15

**Authors:** Yuji Takahashi, Takuto Fujii, Kyosuke Fujita, Takahiro Shimizu, Taiga Higuchi, Yoshiaki Tabuchi, Hisato Sakamoto, Ichiro Naito, Koji Manabe, Shinichi Uchida, Sei Sasaki, Akira Ikari, Kazuhiro Tsukada, Hideki Sakai

**Affiliations:** 1 Department of Pharmaceutical Physiology, Graduate School of Medicine and Pharmaceutical Sciences, University of Toyama, Toyama 930-0194, Japan; 2 Life Science Research Center, University of Toyama, Toyama 930-0194, Japan; 3 School of Medicine, Kitasato University, Sagamihara 228-8555, Japan; 4 Faculty of Medicine, Okayama University, Okayama 700-8558, Japan; 5 Department of Internal Medicine, Shigei Medical Research Hospital, Okayama 701-0202, Japan; 6 Graduate School of Medicine, Tokyo Medical and Dental University, Tokyo 113-8519, Japan; 7 Laboratory of Biochemistry, Gifu Pharmaceutical University, Gifu 501-1196, Japan; 8 Department of Surgery II, Graduate School of Medicine and Pharmaceutical Sciences, University of Toyama, Toyama 930-0194, Japan

**Keywords:** ClC-5, H^+^,K^+^-ATPase, Gastric acid, Tubulovesicle, Parietal cell

## Abstract

It has been reported that chloride–proton exchanger ClC-5 and vacuolar-type H^+^-ATPase are essential for endosomal acidification in the renal proximal cells. Here, we found that ClC-5 is expressed in the gastric parietal cells which secrete actively hydrochloric acid at the luminal region of the gland, and that it is partially localized in the intracellular tubulovesicles in which gastric H^+^,K^+^-ATPase is abundantly expressed. ClC-5 was co-immunoprecipitated with H^+^,K^+^-ATPase in the lysate of tubulovesicles. The ATP-dependent uptake of ^36^Cl^−^ into the vesicles was abolished by 2-methyl-8-(phenylmethoxy)imidazo[1,2-*a*]pyridine-3-acetonitrile (SCH28080), an inhibitor of H^+^,K^+^-ATPase, suggesting functional expression of ClC-5. In the tetracycline-regulated expression system of ClC-5 in the HEK293 cells stably expressing gastric H^+^,K^+^-ATPase, ClC-5 was co-immunoprecipitated with H^+^,K^+^-ATPase, but not with endogenous Na^+^,K^+^-ATPase. The SCH28080-sensitive ^36^Cl^−^ transporting activity was observed in the ClC-5-expressing cells, but not in the ClC-5-non-expressing cells. The mutant (E211A-ClC-5), which has no H^+^ transport activity, did not show the SCH28080-sensitive ^36^Cl^−^ transport. On the other hand, both ClC-5 and its mutant (E211A) significantly increased the activity of H^+^,K^+^-ATPase. Our results suggest that ClC-5 and H^+^,K^+^-ATPase are functionally associated and that they may contribute to gastric acid secretion.

## Introduction

ClC-5 belongs to a sub-branch of CLC family of Cl^−^ channels and transporters that includes ClC-3 and ClC-4 (Jentsch et al., 2005; Jentsch, 2008). Like ClC-3 and ClC-4, ClC-5 is localized mainly in endosomal membranes (Devuyst et al., 1999; Günther et al., 1998). Loss-of-function mutations of ClC-5 lead to Dent's disease, an X-chromosome-linked disease characterized by low molecular weight proteinuria, hyperphosphaturia and hypercalciurea (Lloyd et al., 1996; Wrong et al., 1994). ClC-5 is predominantly expressed in the kidney, and its expression largely overlaps with the vacuolar-type H^+^-ATPase (V-ATPase), which is highly expressed in renal proximal tubules (Günther et al., 1998). The endosomal ClC-5 is essential for proximal tubular endocytosis as evidenced by results with the ClC-5 knock-out mice, a model for Dent's disease (Piwon et al., 2000; Wang et al., 2000).

ClC-5 was previously suggested to be an electrically shunting Cl^−^ channel in early endosomes, facilitating intraluminal acidification (Günther et al., 2003; Piwon et al., 2000). Thereafter, ClC-5 has been characterized as a voltage-dependent Cl^−^/H^+^ exchanger rather than a Cl^−^ channel (Picollo and Pusch, 2005; Scheel et al., 2005; Zifarelli and Pusch, 2009). ClC-5 may compensate the charge accumulation by the endosomal V-ATPase via coupling directly vesicular pH gradients to Cl^−^ gradients as a secondary active ion transporter (Picollo and Pusch, 2005; Scheel et al., 2005). On the other hand, it has been suggested that ClC-5 is directly involved in endosomal acidification by exchanging endosomal Cl^−^ to H^+^ (Smith and Lippiat, 2010); that is, H^+^ transport of ClC-5 is not coupled to that of V-ATPase.

Expression of ClC-5 was also reported in the rat intestinal tissues (duodenum, jejunum, ileum and colon), although its expression level was lower than that in the kidney (Vandewalle et al., 2001). ClC-5 was found to be colocalized with V-ATPase in a vesicle-rich region beneath the apical brush border of enterocytes, suggesting the significant role of ClC-5 in the endocytotic pathways of epithelial intestinal cells (Vandewalle et al., 2001).

So far, it has not been reported about function of ClC-5 in the stomach. Gastric parietal cells secrete hydrochloric acid (HCl) into the lumen of the stomach. On activation of parietal cells, large proportion of the tubulovesicles, which are rich in gastric proton pump (H^+^,K^+^-ATPase), fuse each other and connect with the apical canalicular membrane. The tubulovesicular and apical membranes may not mix but remain separate and distinct in this stimulated phase (Fujii et al., 2009; Nishi et al., 2012). Cystic fibrosis transmembrane conductance regulator (CFTR) Cl^−^ channel (Sidani et al., 2007) and solute carrier 26A9 (SLC26A9), which functions as a Cl^−^ channel and Cl^−^/HCO_3_^−^ exchanger (Xu et al., 2008) in tubulovesicles and K^+^-Cl^−^ cotransporter-4 (KCC4) in apical membranes (Fujii et al., 2009) are reported as a candidate of Cl^−^ transporting molecules for HCl secretion.

In the present study, we have found that ClC-5 is expressed in gastric tubulovesicles and that ClC-5 and H^+^,K^+^-ATPase are functionally associated. Our results suggest the novel function of ClC-5 in gastric acid secretion.

## Results

### Expression of ClC-5 in gastric tubulovesicles

**Fig. 1.**
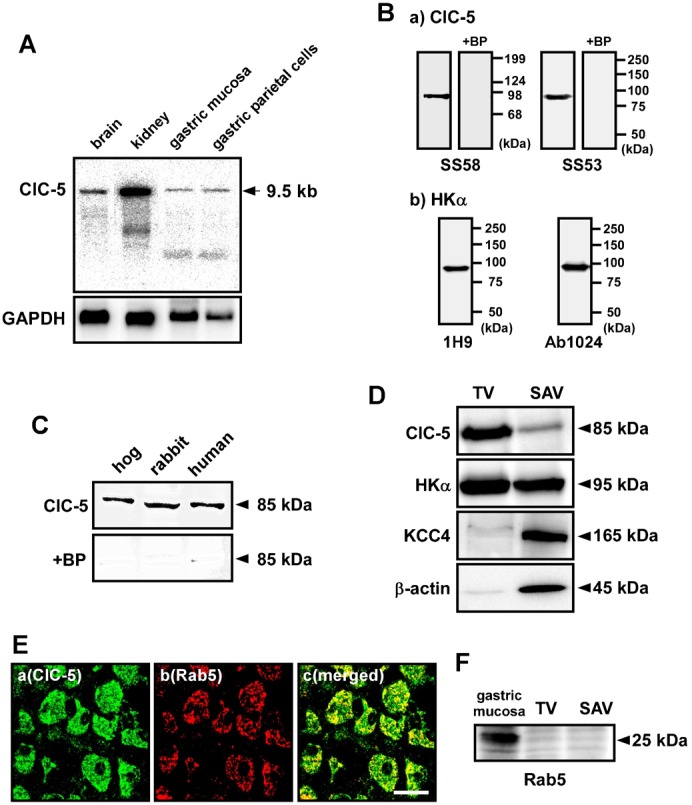
Expression of ClC-5 in gastric samples. (A) Expression of ClC-5 mRNA in the stomach. Northern blotting was performed with poly A^+^ RNA (2.5 µg/lane) from brain, kidney and stomach of rabbits. A single band of 9.5 kb was detected with the ClC-5 cDNA probe. As a control, expression of GAPDH (1.3 kb) was examined. (B) Specificity of anti-ClC-5 and anti-H^+^,K^+^-ATPase α-subunit (HKα) antibodies. Western blotting was performed with hog gastric tubulovesicles (10 µg of protein) by using anti-ClC-5 antibodies (SS58 and SS53) (a) and anti-HKα antibodies (1H9 and Ab1024) (b). A single band of 85 kDa (a) or 95 kDa (b) was detected. The 85-kDa band was disappeared when the ClC-5 antibodies were preincubated with the corresponding blocking peptide (antibody:peptide  =  1:5) (+BP; a). (C) Western blotting was performed with hog and human gastric tubulovesicles and rabbit gastric P3 fraction (5, 50 and 40 µg of protein, respectively) by using anti-ClC-5 antibody (SS58). In each sample, an 85 kDa-band was detected (upper panel), and the band disappeared in the presence of the corresponding blocking peptide (lower panel). (D) Western blotting was performed with hog tubulovesicles (TV) and stimulation-associated vesicles (SAV) (10 µg of protein) by using anti-ClC-5 (SS58), anti-HKα (1H9), anti-KCC4 and anti-β-actin antibodies. ClC-5 (85 kDa) was predominantly expressed in TV, while KCC4 (165 kDa) and β-actin (45 kDa) were predominantly in SAV. HKα (95 kDa) was found in both TV and SAV. (E) a–c show the same tissue under a microscope. Double immunostaining was performed with hog gastric mucosa by using anti-ClC-5 (SS53) plus anti-Rab5 antibodies. Original magnification: ×63. Scale bars, 20 µm. (F) Western blotting was performed with TV, SAV and membrane fraction of gastric mucosa of hogs (30 µg of protein) by using anti-Rab5 antibody. Rab5 (25 kDa) was expressed in the gastric mucosa.

First, we checked expression levels of ClC-5 mRNA in brain, kidney and stomach of rabbits (Fig. 1A). Northern blotting with the ClC-5 cDNA probe gave a single band of 9.5 kb. This size is the same as that of rat kidney ClC-5 (9.5 kb) (Steinmeyer et al., 1995). Expression level of ClC-5 mRNA in the stomach was lower than that in the kidney (Fig. 1A). For detection of ClC-5 protein, two anti-ClC-5 antibodies (SS53 and SS58) were used. The SS53 (Sakamoto et al., 1999) and SS58 (Fig. 4A) were demonstrated to have no cross reactivity to ClC-3 or ClC-4. In Fig. 1B, Western blotting with SS58 and SS53 gave a single band of 85 kDa in hog gastric samples, which contain H^+^,K^+^-ATPase as shown by using two anti-H^+^,K^+^-ATPase α-subunit (HKα) antibodies (1H9 and Ab1024; Fig. 1B). The size of the gastric ClC-5 protein bands (85 kDa) is close to those of ClC-5 proteins observed in the kidneys of rats (83 kDa) (Vandewalle et al., 2001) and mice (85 kDa) (Sakamoto et al., 1999). The specificity of anti-ClC-5 antibodies for the 85-kDa band was confirmed in the presence of the corresponding blocking peptide (Fig. 1B).

Significant expression of ClC-5 protein was observed in gastric samples of hogs, rabbits and humans (Fig. 1C). Gastric H^+^,K^+^-ATPase is abundantly expressed in parietal cells and localized in intracellular tubulovesicles and apical canalicular membrane of the cells (Fujii et al., 2009). In the present study, two types of hog gastric vesicles were prepared; one is intracellular tubulovesicles (TV) and the other is stimulation-associated vesicles (SAV) derived from the apical canalicular membrane. HKα was rich in both TV and SAV (Fig. 1D). β-actin was rich in SAV as previously reported (Fujii et al., 2009). Interestingly, expression level of ClC-5 in TV was much higher than that in SAV (Fig. 1D). This expression pattern of ClC-5 is apparently different from that of KCC4 which is functionally associated with H^+^,K^+^-ATPase in the apical canalicular membrane (Fujii et al., 2009) (Fig. 1D). In hog parietal cells, ClC-5 was found to be localized in both the endosome marker (Rab5)-positive and negative areas (Fig. 1E), suggesting that ClC-5 was expressed in both endosomes and tubulovesicles (TV). In fact, no significant expression of Rab5 was observed in TV (Fig. 1F).

### Localization of ClC-5 in gastric parietal cells at the luminal region of the glands

**Fig. 2.**
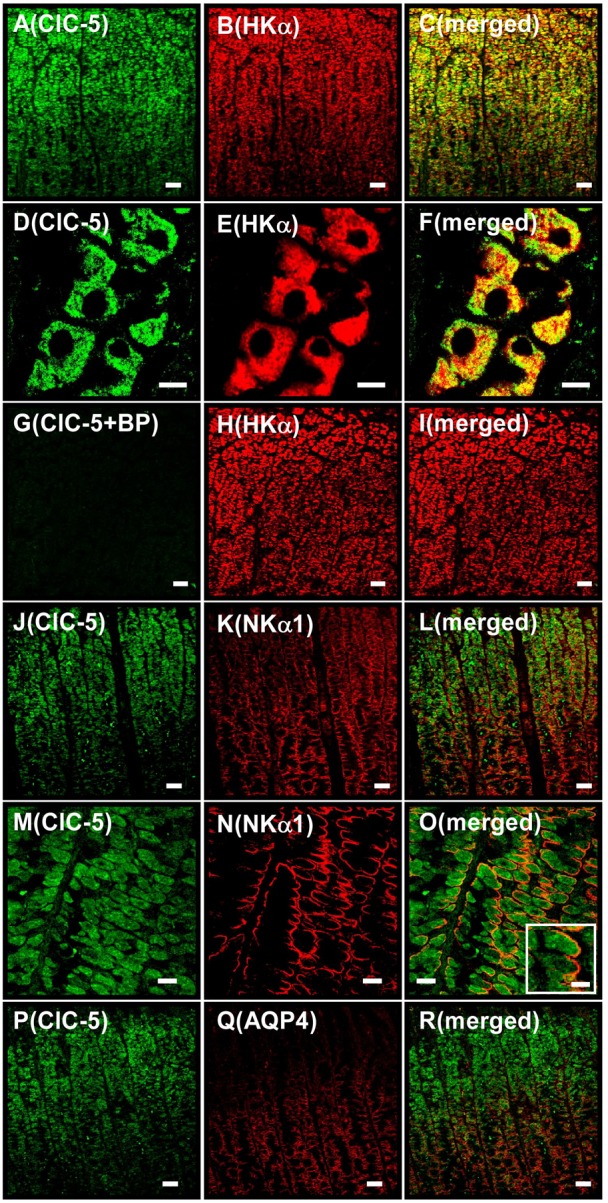
Immunostaining for ClC-5 in isolated hog gastric mucosa. A–C show the same tissue under a microscope (as do D–F, G–I, J–L, M–O and P–R). Double immunostaining was performed with hog gastric mucosa by using anti-ClC-5 (SS53) plus anti-HKα (1H9) antibodies (A–I), anti-ClC-5 plus anti-NKα1 antibodies (J–O), and anti-ClC-5 plus anti-AQP4 antibodies (P–R). (A–F) Localizations of ClC-5 (A and D), HKα (B and E) and ClC-5 plus HKα (merged image; C and F) are shown. (G–I) Anti-ClC-5 antibody was pretreated with the blocking peptide. Localizations of ClC-5 (G), HKα (H) and ClC-5 plus HKα (merged image; I) were shown. Positive ClC-5 staining disappeared (G). (J–O) Localizations of ClC-5 (J and M), NKα1 (K and N) and ClC-5 plus NKα1 (merged image; L and O). In the inset of O, an enlarged image of a parietal cell is shown. (P–R) Localizations of ClC-5 (P), AQP4 (Q) and ClC-5 plus AQP4 (merged image; R). Original magnification: ×20 (A–C, G–L and P–R), ×40 (M–O) and ×63 (D–F). Scale bars, 50 µm (A–C, G–L and P–R), 10 µm (D–F and inset of O), and 20 µm (M–O).

In the immunohistochemistry of isolated hog gastric mucosa, the distribution pattern of ClC-5 mostly accorded with that of HKα (Fig. 2A–F), but not completely overlapped (Fig. 2F). Taking into account the results in Fig. 1, areas exhibited the yellow and red colors in parietal cells (Fig. 2F) are suggested to be intracellular tubulovesicles and the apical canalicular membrane, respectively. The specificity of anti-ClC-5 antibody for positive staining was confirmed by using the blocking peptide (Fig. 2G–I). On the other hand, the distribution of ClC-5 was apparently different from that of Na^+^,K^+^-ATPase α1-subunit (NKα1) which is located in the basolateral membrane of the cells (Fig. 2J–O). It has been reported that younger parietal cells at the luminal region of the glands much more actively secrete acid than older parietal cells at the basal region (Bamberg et al., 1994; Karam et al., 1997; Sachs, 2001). Aquaporin 4 (AQP4) has been reported to be localized in the basolateral membrane of the parietal cells at the basal region of the gastric glands (Carmosino et al., 2001). Interestingly the present double immunostaining of AQP4 and ClC-5 in the gastric mucosa showed that ClC-5 is expressed in the parietal cells more abundantly at the luminal region of the glands than at the basal region (Fig. 2P–R).

### Association of ClC-5 with H^+^,K^+^-ATPase in TV

**Fig. 3.**
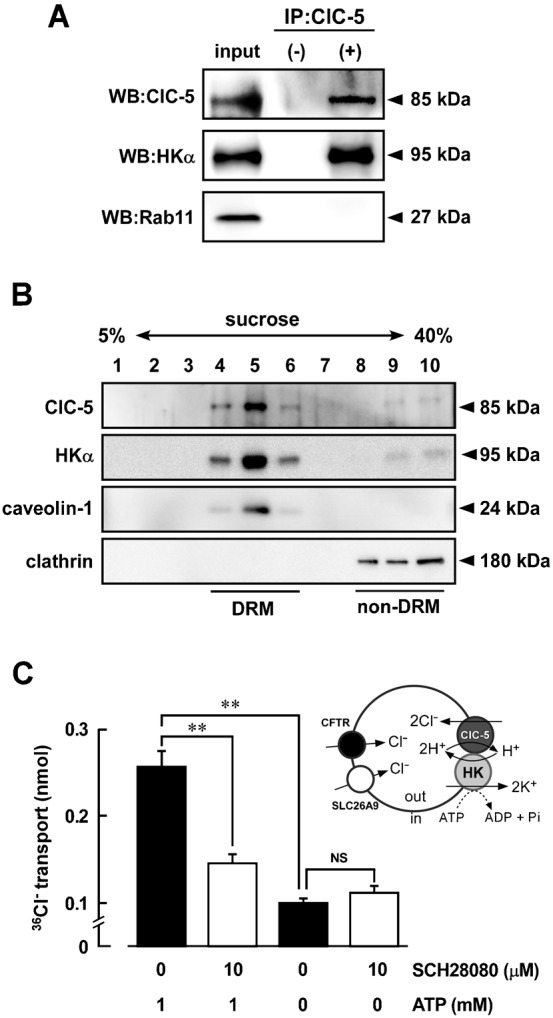
Association of ClC-5 with H^+^,K^+^-ATPase in hog gastric tubulovesicles. (A) Immunoprecipitation (IP) was performed with the detergent extracts of the hog gastric tubulovesicles (100 µg of potein) using anti-ClC-5 antibody (SS58) and protein A/G-agarose (IP: ClC-5, +). In control experiments, preimmune serum instead of the antibody was used (IP: ClC-5, −). The detergent extracts and immunoprecipitation samples were detected by Western blotting (WB) using antibodies for ClC-5 (SS58) labeled with HRP, HKα (1H9) and Rab11. The immunoprecipitation shown is representative of three independent experiments. (B) Detergent-resistant membrane (DRM) fractions and non-DRM fractions were isolated from hog gastric tubulovesicles by sucrose gradient (5–40%) as described in Materials and Methods. Western blotting was performed by using antibodies for ClC-5 (SS53), HKα (Ab1024), caveolin-1 and clathrin. (C) Inhibition of ^36^Cl^−^ uptake into tubulovesicles by an inhibitor of H^+^,K^+^-ATPase (SCH28080). The ^36^Cl^−^ uptake in tubulovesicles was measured as described in Materials and Methods. Effects of 1 mM ATP and/or 10 µM SCH28080 on the ^36^Cl^−^ uptake were examined. As a control, the uptake was measured in the absence of ATP. *n* = 10. NS, not significantly different (*P*>0.05); **, significantly different (*P*<0.01).

To study whether ClC-5 is associated with HKα in TV of hogs, immunoprecipitation was performed with an anti-ClC-5 antibody. The subsequent Western blotting of the immune pellets with anti-ClC-5 and anti-HKα antibodies gave a clear band for ClC-5 (85 kDa) and HKα (95 kDa), respectively (Fig. 3A). In contrast, the blotting with an anti-Rab11 antibody (as a negative control) gave no band for Rab11 (27 kDa) which is present in TV and related to the vesicular trafficking machinery in gastric parietal cells (Calhoun and Goldenring, 1997) (Fig. 3A). These results suggest that ClC-5 and H^+^,K^+^-ATPase are located close together in the TV.

Caveolae are known to be insoluble for treatment with detergents such as Triton X-100 and CHAPS at low temperature and form detergent resistance membrane (DRM) fractions with low density. In the present study, caveolae were isolated from hog TV by using CHAPS and sucrose gradient. Caveolin-1 was used as a marker for caveolae (Rothberg et al., 1992). As shown in Fig. 3B, ClC-5 and HKα were mainly distributed in the DRM fractions, in which caveolin-1 was expressed. In non-DRM fractions, in which clathrin was expressed, no significant distribution of ClC-5 and HKα was observed (Fig. 3B).

Most of TVs are inside-out vesicles (Asano et al., 1987): that is, ATP-binding site of H^+^,K^+^-ATPase faces outside and H^+^ is transported from outside to inside (Fig. 3C). Here we measured ^36^Cl^−^ uptake into TV in the presence of valinomycin, a potassium ionophore. In Fig. 3C, the presence of ATP significantly increased the ^36^Cl^−^ uptake into TV. SCH28080, a specific inhibitor of H^+^,K^+^-ATPase, significantly inhibited the ATP-dependent ^36^Cl^−^ uptake, while no SCH28080-induced inhibition was observed in the absence of ATP (Fig. 3C). These results suggest that H^+^,K^+^-ATPase-coupled secondary active Cl^−^ transporter(s) are present. Although it has been reported the expression of Cl^−^ channels such as CFTR (Fujii et al., 2009) and SLC26A9 (Xu et al., 2008) in TV, these proteins are not secondary active transporters coupled with the ATPase. Since ClC-5 can functionally couple to H^+^,K^+^-ATPase via H^+^ transport, the SCH28080-sensitive ^36^Cl^−^ uptake in TV may be, at least, partly mediated by ClC-5. On the other hand, SCH28080-insensitive ^36^Cl^−^ uptake was also observed (Fig. 3C). This passive Cl^−^ transport may be mediated by CFTR and SLC26A9.

### Stable coexpression of ClC-5 and H^+^,K^+^-ATPase in HEK293 cells

**Fig. 4.**
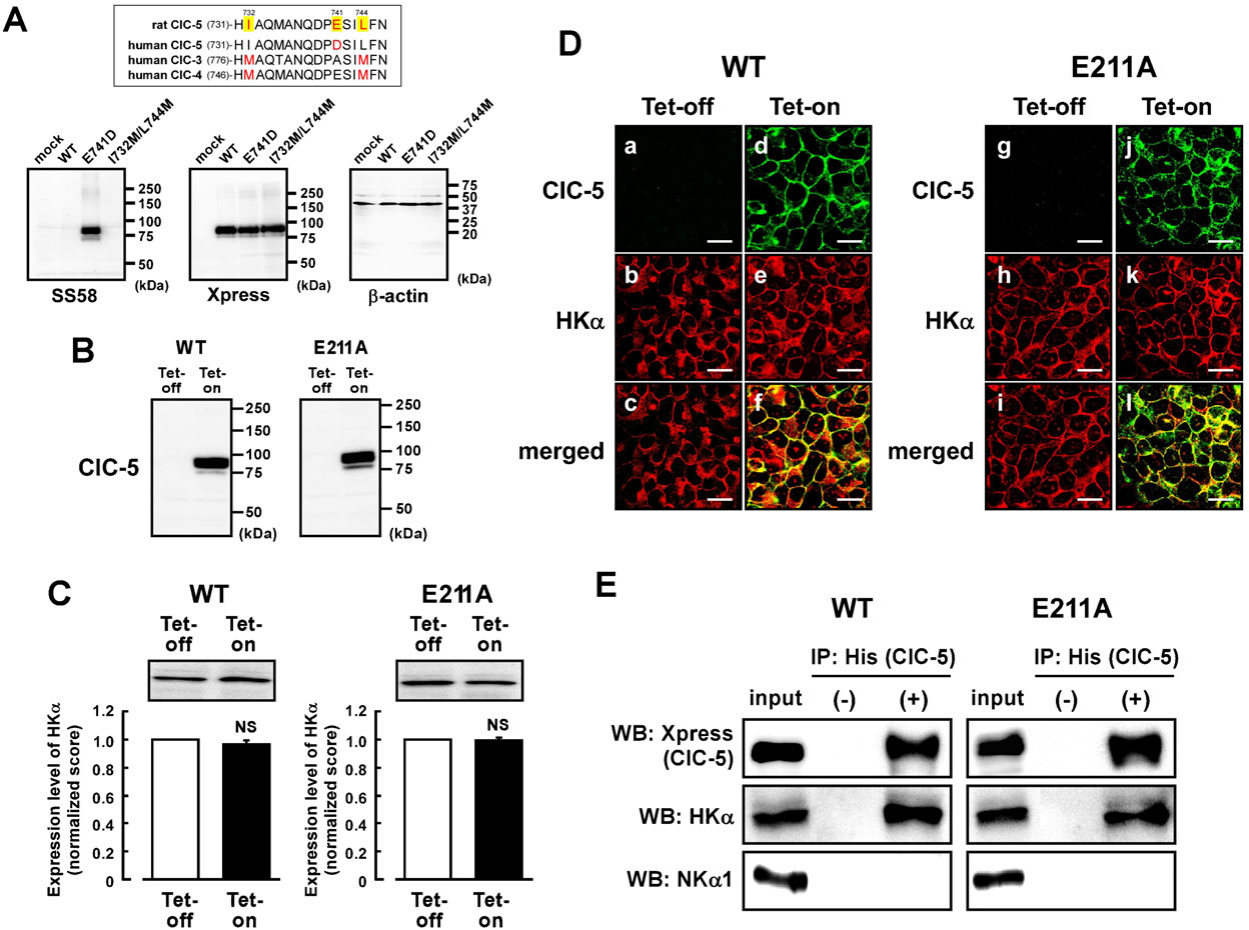
Tetracycline-regulated expression of ClC-5 in the HEK293 cells stably expressing gastric H^+^,K^+^-ATPase. (A) Alignments of rat ClC-5, human ClC-5, human ClC-3 and human ClC-4 around an epitope of the anti-ClC-5 antibody are shown (upper panel). WT-ClC-5, E741D-ClC-5 and I732M/L744M-ClC-5 were transiently transfected in the HEK293 cells. In lower panels, Western blotting was performed with the membrane fraction (50 µg of protein) using anti-ClC-5 (SS58) (left), anti-Xpress (middle) and anti-β-actin (right) antibodies. No significant signal was observed in mock-transfected cells. (B) The tetracycline-regulated expression systems of WT-ClC-5 and E211A-ClC-5 were introduced to the HEK293 cells stably expressing H^+^,K^+^-ATPase. The cells were treated with (Tet-on) or without (Tet-off) 2 µg/ml tetracycline. Expression of WT- and E211A-ClC-5 in the membrane fraction of the cells (30 µg of protein) was confirmed by Western blotting using anti-Xpress antibody. (C) Expression level of HKα in the Tet-on cells was compared with that in the Tet-off cells. In the upper panel, a representative picture of Western blotting is shown. In the lower panel, the quantified score for the Tet-off cells is normalized as 1. *n* = 6. NS, *P*>0.05. (D) a–c show the same cells under a microscope (as do d–f, g–i, j–l). Double immunostaining was performed with the WT Tet-off cells (a–c), WT Tet-on cells (d–f), E211A Tet-off cells (g–i) and E211A Tet-on cells (j–l) using anti-Xpress (for ClC-5) plus anti-HKα (Ab1024) antibodies. Localizations of WT-ClC-5 (a and d), E211A-ClC-5 (g and j) and HKα (b, e, h and k), WT-ClC-5 plus HKα (merged images; c and f), and E211A-ClC-5 plus HKα (merged images; i and l) are shown. Scale bars, 20 µm. (E) WT-ClC-5 (left) and E211A-ClC-5 (right) are assembled to HKα in the HEK293 cells. Immunoprecipitation was performed with the detergent extracts of the Tet-on cells by using anti-His-tag antibody (for ClC-5) and protein A-agarose. The detergent extract (input) and the immunoprecipitation samples obtained with (IP: His(ClC-5), +) and without (IP: His(ClC-5), −) the antibody were detected by Western blotting (WB) using anti-Xpress antibody for detecting ClC-5 (top panel) and anti-HKα antibody (1H9; middle panel) and anti-NKα1 antibody (bottom panel; 100 kDa). In WB, anti-Xpress and anti-HKα antibodies were labeled with horseradish peroxidase. The immunoprecipitation shown is representative of three independent experiments.

It is noted that amino acid sequence of the antigen peptide used for preparation of the anti-hog, rabbit and human ClC-5 antibody (KHIAQMANQDPDSILFN) is slightly different from that of the corresponding region of rat ClC-5 (KHIAQMANQDPESILFN); that is, one amino acid residue is different (E741 in rat ClC-5). Here, rat ClC-5 cDNA was cloned. In Fig. 4A, the ClC-5 antibody (SS58) did not bind to WT-ClC-5 but to E741D-ClC-5 of rats. We also constructed the I732M-L744M double mutant (I732M/L744M-ClC-5) to check whether the SS58 antibody can also react with ClC-3 or ClC-4. The antibody did not bind to the I732M/L744M-ClC-5 (Fig. 4A). These results confirmed again high specificity of the antibody. No significant expression of endogenous human ClC-5 protein was observed in mock-transfected HEK293 cells (Fig. 4A, lower left) in which β-actin was normally expressed (Fig. 4A, lower right). On the other hand, the anti-Xpress antibody binds to WT-, E741D-, and I732M/L744M-ClC-5 because the Xpress sequence is tagged at the N-terminus of the cloned ClC-5 (Fig. 4A, lower middle).

Next, the tetracycline-regulated expression systems of WT-ClC-5 and E211A-ClC-5 which has no H^+^ transport activity (Picollo and Pusch, 2005) were constructed in the HEK293 cells that stably expressing α- and β-subunits of gastric H^+^,K^+^-ATPase. Exogenous expressions of WT- and E211A-ClC-5 were observed in the cells treated with tetracycline (Tet-on cells), but not in the cells treated without tetracycline (Tet-off cells) (Fig. 4B). Expression levels of HKα in the Tet-on cells were not significantly different from those in the Tet-off cells (Fig. 4C). WT- and E211A-ClC-5 and HKα were found to be partially present in the plasma membrane of the Tet-on cells (Fig. 4D). To check whether WT/E211A-ClC-5 and H^+^,K^+^-ATPase are located close together in the Tet-on cells as is the case in the TV (Fig. 3A), immunoprecipitation was performed with an anti-His tag antibody (for ClC-5). The subsequent Western blotting of the immune pellets with an anti-HKα antibody gave a band for HKα (Fig. 4E), suggesting the association between WT/E211A-ClC-5 and HKα. On the other hand, an anti-NKα1 antibody gave no band for endogenous Na^+^,K^+^-ATPase (Fig. 4E).

### H^+^,K^+^-ATPase-dependent Cl^−^ transport by ClC-5 in HEK293 cells

**Fig. 5.**
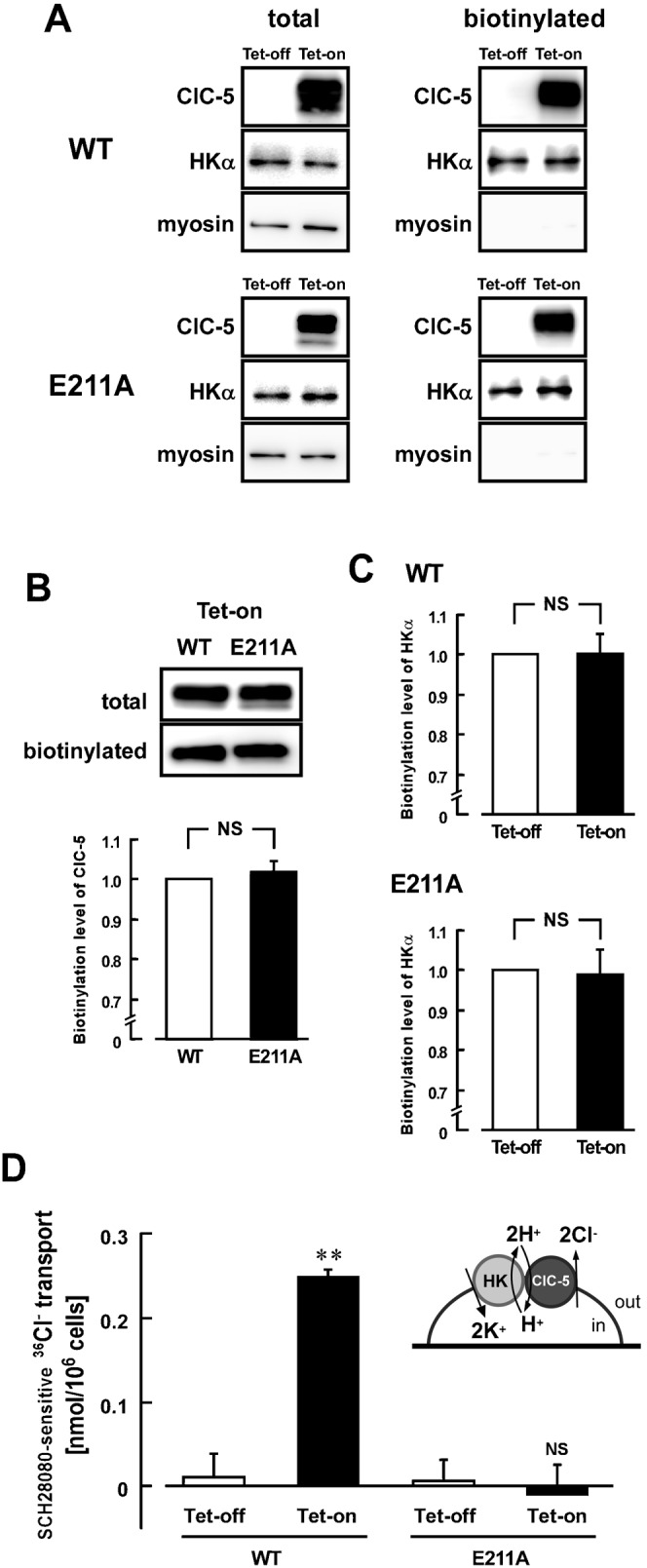
Association of ClC-5 with gastric H^+^,K^+^-ATPase in the HEK293 cells. (A) Cell surface biotinylation was performed with the Tet-on and Tet-off cells of WT-ClC-5 (upper panels) and E211A-ClC-5 (lower panels) prepared as described in Fig. 4. For detecting WT-ClC-5 (upper) or E211A-ClC-5 (lower), HKα and myosin (230 kDa) in the total lysate (left) and the biotinylated fraction (right), anti-Xpress (for ClC-5), anti-HKα (1H9) and anti-myosin antibodies were used, respectively. (B) Amount of biotinylated ClC-5 in the WT Tet-on cells is compared with that of biotinylated E211A-ClC-5 in the E211A Tet-on cells. In the upper panel, representative pictures of Western blotting are shown. In the lower panel, the biotinylated level was estimated by using the following equation: Calibrated biotinylation level of WT-ClC-5 (WT) or E211A-ClC-5 (E211A)  =  (amount of WT or E211A protein in the biotinylated fraction)/(amount of WT or E211A protein in the total lysate). The score for the WT cells is normalized as 1. *n* = 6. NS, *P*>0.05. (C) Amount of biotinylated HKα in the Tet-on cells is compared with that of the Tet-off cells (upper, WT; lower, E211A). The quantified score for the Tet-off cells is normalized as 1. *n* = 6. NS, *P*>0.05. (D) H^+^,K^+^-ATPase-dependent ^36^Cl^−^ transporting activity in the cells. ^36^Cl^−^ was loaded into the Tet-on and Tet-off cells of WT-ClC-5 and E211A-ClC-5 as described in Materials and Methods. The ^36^Cl^−^ transport activity was measured in the presence and absence of 10 µM SCH28080, and the SCH28080-sensitive (H^+^,K^+^-ATPase-dependent) transport activity was calculated. Significant activity was observed only in the WT Tet-on cells. *n* = 4–6. NS, not significantly different (*P*>0.05); **, significantly different (*P*<0.01).

To assess function of WT- and E211A-ClC-5 in the HEK293 cells, it is necessary to know expression levels of ClC-5 protein in the plasma membrane. Therefore, the cell surface biotinylation assay was performed with the cells. As control experiments, expression of myosin, an intracellular protein, was checked (Fig. 5A). In the Tet-on cells, biotinylated level of WT-ClC-5 was not significantly different from that of E211A-ClC-5 (Fig. 5B). Furthermore, biotinylated level of HKα in the Tet-on cells was not significantly different from that in the Tet-off cells (Fig. 5A,C). Expression level of ClC-5 in the plasma membrane was estimated to be about 15% of total ClC-5 expressed in the HEK293 cells.

Next, we estimated Cl^−^ transport activities of WT- and E211A-ClC-5 in the Tet-on and Tet-off cells. The SCH28080-sensitive ^36^Cl^−^ transport activity in the WT Tet-on cells was significantly greater than that in the WT Tet-off cells (Fig. 5D). In contrast, no activity of the SCH28080-sensitive ^36^Cl^−^ transport was observed in the E211A Tet-on cells (Fig. 5D). It is noted that amounts of incorporated ^36^Cl^−^ before starting the transport experiments were not significantly different in these Tet-on and Tet-off cells (not shown). These results suggest that the ClC-5-derived ^36^Cl^−^ transport depends on the H^+^,K^+^-ATPase activity, and that ClC-5 can functionally couple to gastric H^+^,K^+^-ATPase via H^+^ transport in the HEK293 cells.

### Upregulation of H^+^,K^+^-ATPase activity by coexpression of ClC-5

**Fig. 6.**
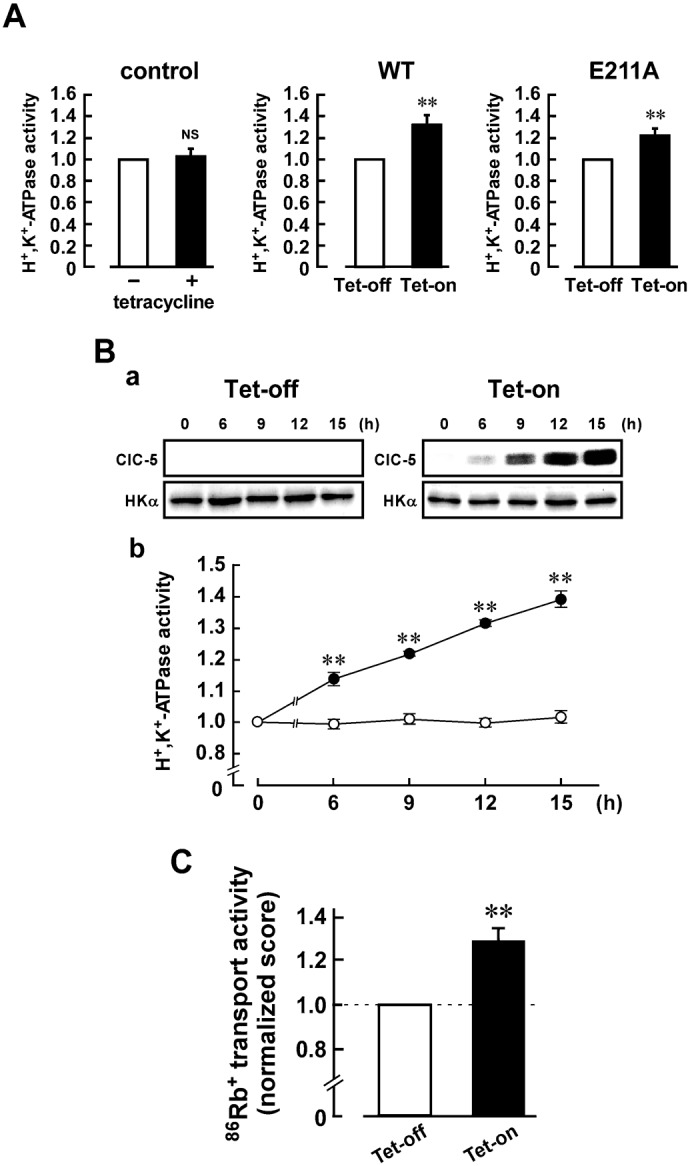
Upregulation of the H^+^,K^+^-ATPase activity in the ClC-5-expressing cells. (A) H^+^,K^+^-ATPase activity in the control HEK293 cells (left panels), and Tet-on and Tet-off cells of WT-ClC-5 (middle panels) and E211A-ClC-5 (right panels). The cells were treated with 2 µg/ml tetracycline for 12 h to obtain the Tet-on cells. The activity in the Tet-on cells was estimated by using the following equation: Calibrated H^+^,K^+^-ATPase activity = [(H^+^,K^+^-ATPase activity in the Tet-on cells)/(level of protein expression of HKα in the Tet-on cells)]/[(H^+^,K^+^-ATPase activity in the Tet-off cells)/(level of protein expression of HKα in the Tet-off cells)]. The H^+^,K^+^-ATPase activity of the WT and E211A Tet-off cells was 0.21±0.03 and 0.24±0.02 µmol Pi/mg of protein/h, respectively (*n* = 6). Calibrated H^+^,K^+^-ATPase activity of the Tet-off cells is normalized as 1. *n* = 6. **, *P*<0.01. (B) The upregulatory effect depends on the expression level of ClC-5 protein. The WT cells were treated with (Tet-on) and without (Tet-off) 2 µg/ml tetracycline for various period (6, 9, 12 and 15 h). In a, expression levels of ClC-5 and HKα proteins in the Tet-on (right) and Tet-off (left) cells are shown. In b, the H^+^,K^+^-ATPase activity of each sample was estimated as in Fig. 6A, and the data were shown as means ± s.e.m. (•, Tet-on; ○, Tet-off). *n* = 6. **, significantly different (*P*<0.01) compared with Tet-off. (C) ^86^Rb^+^ transport activity of the WT Tet-on and Tet-off cells. The ^86^Rb^+^ transport activity was measured in the presence and absence of 50 µM SCH28080, and the SCH28080-sensitive (H^+^,K^+^-ATPase-dependent) transport activity was calculated. The score was calculated by using the following equation: Normalized ^86^Rb^+^ transport activity = (the activity in the Tet-on cells)/(the activity in the Tet-off cells). The ^86^Rb^+^ transport activity of the Tet-off cells was 0.34±0.07 nmol ^86^Rb^+^/min/10^6^ cells (*n* = 5). The score for Tet-off cells is normalized as 1. *n* = 5. **, *P*<0.01.

Interestingly, the SCH28080-senstive K^+^-ATPase activity (H^+^,K^+^-ATPase activity) in the Tet-on cells was significantly greater than that in the Tet-off cells (Fig. 6A, middle), although the total expression level (Fig. 4C) and biotinylated level (Fig. 5C) of HKα in the Tet-on cells were not significantly different from those in the Tet-off cells. It is noted that expression of E211A-ClC-5 also increased the H^+^,K^+^-ATPase activity (Fig. 6A, right). Treatment of tetracycline had no significant effects on the H^+^,K^+^-ATPase activity in the control HEK293 cells (Fig. 6A, left). The upregulatory effect in the Tet-on cells depended on the expression level of ClC-5 (Fig. 6B). Corresponding to these results, the SCH28080-sensitive ^86^Rb^+^ transport activity in the Tet-on cells was significantly greater than that in the Tet-off cells (Fig. 6C).

## Discussion

In the present study, we found the following. 1) ClC-5 protein is expressed partially in intracellular tubulovesicles of gastric parietal cells. 2) ClC-5 is associated with H^+^,K^+^-ATPase in tubulovesicles, and both of them are highly localized in caveolae. 3) In tubulovesicles, an H^+^,K^+^-ATPase inhibitor suppresses the secondary active Cl^−^ transport. 4) In the HEK293 cells stably expressing ClC-5 and H^+^,K^+^-ATPase, the ClC-5-derived ^36^Cl^−^ transport was suppressed by the H^+^,K^+^-ATPase inhibitor. 5) ClC-5 increases the H^+^,K^+^-ATPase activity in the membrane fraction of the cells stably expressing these proteins.

Previously, it has been reported that ClC-5 mRNA (Sakamoto et al., 1996) and protein (Günther et al., 1998) are not detected in the rat stomach. This may be due to low expression level of ClC-5 in the stomach, compared with predominant expression of it in the kidney (Günther et al., 1998; Sakamoto et al., 1996). In fact, expression level of ClC-5 mRNA in the gastric parietal cells was considerably lower than that in the kidney (Fig. 1A). In *Xenopus laevis* stomach, lower but detectable amounts of ClC-5 mRNA were found (Lindenthal et al., 1997).

ClC-5 protein was expressed in the parietal cells more abundantly at the luminal region of the glands than at the basal region. Gastric parietal cells migrate from the luminal to the basal region of the glands. It has been suggested that the luminal parietal cells more actively secrete acid than do the basal parietal cells (Bamberg et al., 1994; Karam et al., 1997; Sachs, 2001). Therefore, ClC-5 is suggested to be involved in the mechanism of gastric acid secretion. KCC4 was also found to be predominantly expressed in the luminal region (Fujii et al., 2009).

Function of tubulovesicles dramatically changes between resting and stimulated phases in gastric parietal cells. In resting parietal cells, tubulovesicles are present in intracellular compartments underlying the apical canalicular membrane. Upon stimulation, the tubulovesicles fuse each other and connect with the canalicular membrane, resulting in massive acid secretion (Fujii et al., 2009; Nishi et al., 2012). So far, several Cl^−^ channels and transporters such as CFTR (Sidani et al., 2007), SLC26A9 (Xu et al., 2008), CLIC-6 (Nishizawa et al., 2000; Sachs et al., 2007) and KCC4 (Fujii et al., 2009) have been suggested as candidates that could be involved in the luminal Cl^−^ efflux for gastric acid (HCl) secretion. CFTR is localized predominantly in the tubulovesicles (Fujii et al., 2009). SLC26A9 is also expressed in tubulovesicles and plays an essential role in HCl secretion by regulating Cl^−^ secretion and/or by affecting the viability of tubulovesicles/secretory canaliculi in parietal cells (Xu et al., 2008). CLIC-6 is distributed throughout the cytosol (Nishizawa et al., 2000). KCC4 is expressed in the apical canalicular membrane and suggested to contribute to basal HCl secretion in resting parietal cells (Fujii et al., 2009). In the present study, we have found that ClC-5 is expressed in tubulovesicles, suggesting that ClC-5 is involved in massive acid secretion in stimulated parietal cells. In future, it will be necessary to clarify whether CFTR, SLC26A9 and ClC-5 are expressed in all tubulovesicles or in part of tubulovesicles if further separation of tubulovesicles into different subtypes becomes possible. It will be also important to examine the expression and function of other ClC members such as ClC-3 and ClC-4 in parietal cells.

So far, there have been no reports that focus on the morphology and function of stomach in the ClC-5-deficient mice and the Dent's disease patients. This probably means that no severe gastric failures occur in these mice and patients. It is noted that no difference in the character of gastric secretion was found between WT- and CFTR-deficient mice (McDaniel et al., 2005). Sidani et al. suggested that complete ablation of the CFTR gene may potentially cause activation of compensatory mechanisms such as up-regulation of non-CFTR regulated K^+^ channels (Sidani et al., 2007). They found that ΔF508 mutation of CFTR caused impaired gastric acid secretion in mice (Sidani et al., 2007).

ClC-5 and V-ATPase are present in renal endosome and functional relation between the two proteins has been discussed. At present, the following two mechanisms are postulated. 1) ClC-5 transports two Cl^−^ ions into the vesicle lumen in exchange for a single proton. This provides an anion shunt for the pumping of protons into endosomes by V-ATPase since the 2Cl^−^ to 1H^+^ gives a net influx of 3 negative charges (Smith and Lippiat, 2010; Zifarelli and Pusch, 2009). In addition, endosomal Cl^−^ concentration, which is raised by ClC-5 in exchange for protons accumulated by V-ATPase, may play a role in renal endocytosis (Novarino et al., 2010). 2) When ClC-5 exchanges two Cl^−^ ions from the endosomal lumen for a proton from the cytoplasm, it can directly acidify the endosome in paralleled with V-ATPase. This reversal mode of ClC-5 operation would be limited to the newly formed endocytic vesicle, where the transient interior-negative Donnan potential may drive extrusion of endocytosed Cl^−^ through ClC-5 in exchange for H^+^, but the compensatory shunt conductance has not yet been identified (Smith and Lippiat, 2010).

Here, we found that ClC-5 and H^+^,K^+^-ATPase are functionally associated. In gastric tubulovesicles, H^+^,K^+^-ATPase-dependent (SCH28080-inhibitable) transport of Cl^−^ into the vesicle lumen was observed (Fig. 3C). Furthermore, the H^+^,K^+^-ATPase-dependent Cl^−^ transport was observed in the ClC-5-expressing cells but not in ClC-5-non-expressing cells and E211A-ClC-5-expressing cells (Fig. 5D). ClC-5 and H^+^,K^+^-ATPase transport 2Cl^−^/H^+^ and 2H^+^/2K^+^, respectively. Therefore, HCl might be secreted via H^+^ coupling between them.

At present, it is unknown whether ClC-5 is involved in endocytosis of tubulovesicles in the gastric parietal cells. In tubulovesicles, Cl^−^/HCO_3_^−^ exchanger PAT1 (SLC26A6) has been suggested to contribute to neutralization of H^+^ and removal of Cl^−^ from secretory membranes during the passage of parietal cells from the stimulated phase to the resting phase (Petrovic et al., 2002).

Recently, we suggested that KCC4 indirectly increases the H^+^,K^+^-ATPase activity by effectively supplying K^+^ to the luminal surface of this ATPase. KCC4 did not activate the ATPase activity in cell-free conditions (Fujii et al., 2009). These findings are interesting from the point of view that the ion transport by the secondary active transporter (KCC4) upregulates the primary active pump activity (H^+^,K^+^-ATPase). On the other hand, ClC-5 increased the H^+^,K^+^-ATPase activity even in the cell-free condition. E211A-ClC-5 also increased the ATPase activity (Fig. 6). These results suggest that ClC-5 directly upregulates H^+^,K^+^-ATPase without coupling of ion transport between these two proteins. In future, it is necessary to clarify how ClC-5 is molecularly associated with H^+^,K^+^-ATPase.

In conclusion, ClC-5 is expressed in the tubulovesicules of gastric parietal cells, and its Cl^−^ transport may be regulated by H^+^,K^+^-ATPase. It is interesting that ClC-5 is functionally associated with two types of proton pump; V-ATPase in the kidney and H^+^,K^+^-ATPase in the stomach.

## Materials and Methods

### Chemicals

HEK293 cells stably expressing α- and β-subunits of gastric H^+^,K^+^-ATPase were established as previously described (Kimura et al., 2002). Ab1024 (Asano et al., 1994) and 1H9 (Medical & Biological Laboratories Co., Nagoya, Japan) were used as the anti-H^+^,K^+^-ATPase α-subunit (HKα) antibodies. Anti-ClC-5 monoclonal antibodies (SS53 and SS58) were prepared as described elsewhere (Sakamoto et al., 1999). The synthetic peptide corresponding to amino acids 730–746 (KHIAQMANQDPDSILFN) in the C-terminus of ClC-5 was purchased from Takara Bio Inc. (Otsu, Shiga, Japan), and used for checking specificity of the anti-ClC-5 antibodies. Anti-KCC4 antibody was prepared as previously described (Fujii et al., 2009). Platinum Taq DNA Polymerase High Fidelity, Lipofectamine 2000, anti-Xpress antibody, Alexa Fluor 488-conjugated anti-mouse, anti-rabbit and anti-goat IgG antibodies and Alexa Fluor 546-conjugated anti-rabbit and anti-rat IgG antibodies were obtained from Invitrogen (Carlsbad, CA, USA). Anti-Na^+^,K^+^-ATPase α1-subunit (NKα1), anti-AQP4 (H-19) and anti-caveolin-1 antibodies and protein A/G-agarose beads were from Santa Cruz Biotechnology (Santa Cruz, CA, USA). Immobilized Protein A and sulfo-NHS-ss-biotin were from Pierce (Rockford, IL, USA). Anti-β-actin, anti-Rab5 and anti-myosin antibodies, avidin and SCH28080 were purchased from Sigma–Aldrich (St Louis, MO, USA). Anti-clathrin heavy chain (X22) antibody was from Affinity BioReagents (Golden, CO, USA). Anti-Rab11 antibody was from Cell Signaling Technology Japan (Tokyo, Japan). All other reagents were of the molecular biology grade or the highest grade of purity available.

### Northern blotting

Poly A^+^ RNAs of tissue samples were prepared by using Poly ATtract mRNA isolation system II (Promega, Madison, WI, USA). The amplified products were sequenced and used for preparation of the ^32^P-labeled cDNA probes. The rabbit ClC-5 probe was 305 bp long and corresponded to nucleotides 499–803 of the ClC-5 cDNA. The rabbit GAPDH probe was 493 bp long and corresponded to nucleotides 443–935 of the GAPDH cDNA. For Northern blot analysis, poly A^+^ RNA of each sample (2.5 µg) was separated on a 1% agarose/formaldehyde gel and transferred onto a nylon membrane (Zeta-probe GT, Bio-Rad). The membrane was hybridized with the ^32^P-labelled cDNA fragment of ClC-5 or GAPDH, and exposed to the Imaging Plate (Fuji Film) for 6 h (GAPDH) or 48 h (ClC-5).

### Preparation of gastric samples

Animals were humanely killed in accordance with the guidelines presented by the Animal Care and Use Committee of University of Toyama. Human gastric specimens were obtained from surgical resection of a Japanese patient with gastric cancer (70 years, male) in accordance with the recommendations of the Declaration of Helsinki. Informed consents were obtained from the patient at University of Toyama. The normal gastric mucosae used for the experiments were 10–20 cm apart from the carcinoma. Hog and human gastric tubulovesicles (TV) (Fujii et al., 2009), hog stimulation-associated vesicles (SAV) (Fujii et al., 2009) and rabbit gastric P3 fraction (Hori et al., 2004) rich in H^+^,K^+^-ATPase were prepared as described previously.

### Preparation of membrane fractions

For preparing membrane fractions of HEK293 cells, the cells were incubated in low ionic salt buffer (0.5 mM MgCl_2_ and 10 mM Tris-HCl, pH 7.4) supplemented with the protease inhibitor cocktail (10 µg/ml aprotinin, 10 µg/ml phenylmethylsulfonyl fluoride, 1 µg/ml leupeptin and 1 µg/ml pepstatin A) at 0°C for 10 min. Subsequently, they were homogenized with 40 strokes in a Dounce homogenizer, and the homogenate was diluted with an equal volume of a solution containing 500 mM sucrose and 10 mM Tris-HCl (pH 7.4). The cell suspension was homogenized with 40 more strokes. The homogenized suspension was centrifuged at 800 × *g* for 10 min. The supernatant was centrifuged at 100,000 × *g* for 90 min, and the pellet was suspended in a solution containing 250 mM sucrose and 5 mM Tris-HCl (pH 7.4).

### Isolation of detergent resistant membrane (DRM)

The membrane proteins were lysed with ice-cold MBS buffer (150 mM NaCl and 25 mM MES-NaOH, pH 6.5) containing 1% CHAPS and the protease inhibitor cocktail for 15 min. The solution was mixed with equal volume of 66% sucrose in MBS buffer, the mixture was placed at the bottom of an ultracentrifuge tube, and a discontinuous gradient was formed by overlaying with the 30% sucrose and 5% sucrose solutions. The sample was centrifuged at 261,000 × *g* in SW41Ti rotor (Beckman) for 18 h at 4°C. Ten fractions of 1 ml each were collected from the top of the gradient, and proteins were precipitated by acetone before SDS-polyacrylamide gel electrophoresis and Western blotting. The DRM fractions were rich in caveolin-1, a marker of caveolae (Fig. 3B).

### Western blotting

Western blotting was carried out as described previously (Sakai et al., 2004). The signals were visualized with ECL system (GE Healthcare) or West Femto Maximum Sensitivity Substrate (Thermo Fisher Scientific). To quantify the chemiluminescence signals on the membranes, a FujiFilm LAS-1000 system and the Multi Gauge software (Fuji Film) were used. Anti-HKα antibodies were used at 1:5,000 (1H9) and 1:3,000 dilution (Ab1024). Anti-ClC-5 antibodies (SS53 and SS58) were used at 1:2,000 dilution. For the negative control, 1 volume of the anti-ClC-5 antibody was preincubated with 5 volumes of the corresponding blocking peptide. Anti-KCC4, anti-Rab11, anti-β-actin and anti-Rab5 antibodies were used at 1:1,000 dilution. Anti-caveolin-1 and anti-clathrin heavy chain antibodies were used at 1:2,000 dilution. Anti-Xpress and anti-myosin antibodies were used at 1:5,000 dilution. Anti-NKα1 antibody was used at 1:10,000 dilution. When indicated, anti-ClC-5 (SS58), anti-Xpress and anti-HKα antibodies were labeled with horseradish peroxidase (HRP) using Peroxidase Labeling Kit-NH_2_ (Dojindo Laboratories, Kumamoto, Japan).

### Immunohistochemistry

The gastric mucosa isolated from hog stomach was fixed in PLP (Periodate-Lysine-Paraformaldehyde) containing 10 mM sodium periodate, 75 mM lysine and 2% paraformaldehyde for 12 h at 4°C. The PLP-fixed mucosa were incubated with a series of PBS containing 5% sucrose (for 4 h), 10% sucrose (for 4 h), 15% sucrose (for 4 h) and 20% sucrose (for 12 h) at 4°C. The mucosa embedded in the optimum cutting temperature compound (Sakura Finetechnical Co., Tokyo, Japan) and was cut at 6 µm. The sections were pre-treated with 3% BSA (in PBS) for 1 h at room temperature to prevent nonspecific binding of antibodies. Subsequently, the sections were incubated with anti-ClC-5 (SS53; 1:50 dilution), anti-HKα (Ab1024; 1:100 dilution), anti-Rab5 (1:100 dilution), anti-NKα1 (1:100 dilution), or anti-AQP4 antibody (1:100 dilution) for 15 h at 4°C. Alexa Fluor 488-conjugated and Alexa Fluor 546-conjugated anti-IgG antibodies (1:100 dilution) were used as secondary antibodies. Immunofluorescence images were visualized using a Zeiss LSM 510 laser scanning confocal microscope.

### Measurement of ^36^Cl^−^ transport in gastric tubulovesicles

Hog gastric tubulovesicles (100 µg of protein) were incubated with a solution containing 250 mM sucrose, 15 mM KCl, 3 mM MgSO_4_, 1 mM ATP, 20 µM ouabain, 10 µM *R*-(+)-[(2-*n*-butyl-6,7-dichloro-2-cyclopentyl-2,3-dihydro-1-oxo-1*H*-inden-5-yl)oxy] acetic acid (DIOA), 10 µM furosemide and 100 mM PIPES-Tris (pH 7.4) for 2 min at 37°C. The solution was supplemented with and without 10 µM SCH28080, a specific inhibitor of gastric H^+^,K^+^-ATPase. Then, 5 µCi/ml H^36^Cl and 10 µg/ml valinomycin were added to reaction mixtures, and they were incubated for 5 min at 37°C. The samples were rapidly filtered through a 0.45-µm HAWP filter (Millipore Co., Bedford, MA, USA). To calibrate nonspecific binding of H^36^Cl to the vesicles and the filter, the experiment was performed in the absence of ATP. The filter was washed with a solution containing 5 mM KCl, 250 mM sucrose and 100 mM PIPES-Tris (pH 7.4); transferred to a counting vial; and solubilized with 5 ml of ACS II scintillant. Then the radioactivity of ^36^Cl^−^ was measured.

### Plasmid construction

A full-length cDNA encoding rat ClC-5 was inserted into the pcDNA4/His vector containing the Xpress tag sequence at N-terminal side (Invitrogen) by using *Bam*HI and *Not*I restriction sites (ClC-5-pcDNA4/His vector). The cDNA encoding Xpress-tagged ClC-5 from the pcDNA4/His was introduced into the pcDNA5/TO vector by using *Afl*II and *Not*I restriction sites (ClC-5-pcDNA5/TO vector). Site-directed mutagenesis for preparing the E211A (E211A-ClC-5-pcDNA5/TO vector), E741D (E741D-ClC-5-pcDNA4/His vector), and I732M/L744M (I732M/L744M-ClC-5-pcDNA4/His vector) mutants were performed using the QuikChange II site-directed mutagenesis kit (Stratagene) and appropriate primers (E211A, sense: gagcctgggtaaagcgggccccctagtgc and anti-sense: gcactagggggcccgctttacccaggctc; E741D, sense: tggcaaaccaagaccccgattccattctcttcaa and anti-sense: ttgaagagaatggaatcggggtcttggtttgcca; I732M, sense: caaaaaggatgtgttaaagcacatggcgcagatggcaaa and anti-sense: tttgccatctgcgccatgtgctttaacacatcctttttg; L744M, sense: ccaagaccccgagtccattatgttcaactagaagcatggg and anti-sense: cccatgcttctagttgaacataatggactcggggtcttgg). The mutated cDNA sequences were verified using an ABI PRISM 310 sequencer (Applied Biosystems).

### Expression of ClC-5 in HEK293 cells

For establishing the tetracycline-regulated expression system of ClC-5 in the HEK293 cells stably expressing gastric H^+^,K^+^-ATPase α- and β-subunits, the cells were cotransfected with the ClC-5-pcDNA5/TO or E211A-ClC-5-pcDNA5/TO plus pcDNA6/TR vectors (Invitrogen) using Lipofectamine 2000, and cultured in D-MEM supplemented with 10% FBS for 24 h. Then, the transfected cells were selected in the presence of 0.4 mg/ml hygromycin B (Wako Pure Chemical Industries, Osaka, Japan), 7 µg/ml blasticidin S (Kaken Pharmaceutical Co., Tokyo, Japan), 0.25 mg/ml G418 (Enzo Life Sciences) and 0.1 mg/ml zeocin (Invitrogen). To check the tetracycline-regulated ClC-5 expression, each cell line was treated with 2 µg/ml tetracycline (Invitrogen) for 12 h. The expression of ClC-5 was confirmed by immunocytochemistry and Western blotting.

### Immunocytochemistry

The HEK293 cells were fixed in ice-cold methanol for 7 min at room temperature, and the cells were permeabilized with 0.3% Triton X-100 and 0.1% BSA (in PBS) for 15 min. Then, the samples were pre-treated with the GSDB buffer (20 mM phosphate buffer (pH 7.4), 450 mM NaCl, 16.7% goat serum and 0.3% Triton X-100) for 30 min, and incubated with anti-Xpress (1:100 dilution) and anti-HKα (Ab1024; 1:100 dilution) antibodies for 60 min at 25°C. Alexa Fluor 546-conjugated IgG and Alexa Fluor 488-conjugated IgG antibodies were used as secondary antibodies (1:100 dilution).

### Immunoprecipitation

Hog tubulovesicles (100 µg of protein) and membrane fractions from HEK293 cells (0.5–2 mg of protein) were lysed with 0.5–1 ml of the solution containing 0.5% Triton X-100 (for vesicles) or 1% Nonidet P-40 (for membrane fractions), 150 mM NaCl, 0.5 mM EDTA and 50 mM Tris-HCl (pH 7.4) for 1 h on ice. The lysate was centrifuged at 100,000 × *g* for 30 min at 4°C. The supernatant was pre-cleared with protein A/G-agarose beads (for vesicles) or protein A-agarose beads (for membrane fractions) for 5 h at 4°C, and reacted with anti-ClC-5 antibody SS58 (for vesicles) or anti-His tag antibody (for membrane fractions) for 15 h at 4°C. The sample was then incubated with protein A/G-agarose (for vesicles) or protein A-agarose (for membrane fractions) beads for 4–6 h at 4°C. The beads were washed and eluted into 250 mM Tris-HCl (pH 6.8) supplemented with 8% SDS, 4% glycerol and 10% β-mercaptoethanol. After centrifugation, the supernatant was used for Western blotting.

### Cell surface biotinylation

Cell surface biotinylation was performed as described previously (Wang et al., 2005). HEK293 cells on 6-well collagen-coated plates were treated with 0.5 mg/ml sulfo-NHS-ss-biotin for 30 min at 4°C. Then, the cells were lysed with 500 µl of the solution containing 1% Triton X-100, 150 mM NaCl, 0.5 mM EDTA and 50 mM Tris-HCl (pH 7.4). The lysate was centrifuged at 15,000 × *g* for 20 min at 4°C. The supernatant (1 mg of protein) was rocked with 40 µl of avidin–agarose beads for 4 h at 4°C. Thirty µg of lysate protein was used to determine the total expression. The beads were washed and eluted into the Laemmli sample buffer. The eluted sample was used for Western blotting.

### Measurement of ^36^Cl^−^ transport in ClC-5 Tet-on and Tet-off cells

The cells were seeded on a collagen type I-coated 6-well culture plate (1.5×10^6^ cells per well) and cultured for 12 h in the D-MEM supplemented with 10% FBS followed by additional 12 h-incubation in the presence of 2 µg/ml tetracycline (for Tet-on cells) and 2 µCi/ml H^36^Cl. In the case for Tet-off cells, tetracycline was omitted. Subsequently, the ^36^Cl^−^-loaded cells were washed and incubated in the reaction medium containing 129 mM sodium gluconate, 15 mM NaCl, 1 mM KCl, 0.5 mM MgSO_4_, 0.5 mM CaCl_2_, 20 µM ouabain, 10 µM DIOA, 10 µM furosemide and 5 mM HEPES-NaOH (pH 7.4) for 3 min at 25°C. To assess the H^+^,K^+^-ATPase-dependent Cl^−^ transport, the experiment was performed in the absence and presence of 10 µM SCH28080. Then, the cells were washed and lysed with 2 ml of the solution containing 150 mM NaCl, 0.5 mM EDTA, 1% Nonident P-40 and 50 mM Tris-HCl (pH 7.4). The samples were solubilized with 5 ml of ACS II scintillant and the radioactivity of ^36^Cl^−^ was measured.

### H^+^,K^+^-ATPase assay

K^+^-stimulated ATPase (K^+^-ATPase) activity was measured in 1 ml of solution comprising 50 µg of membrane protein, 3 mM MgCl_2_, 1 mM ATP, 5 mM NaN_3_, 2 mM ouabain, 15 mM KCl and 40 mM Tris-HCl (pH 6.8), in the presence and absence of 50 µM SCH28080. After incubation for 30 min at 37°C, the inorganic phosphate released was measured as described elsewhere (Yoda and Hokin, 1970). The SCH28080-sensitive K^+^-ATPase activity, defined as the H^+^,K^+^-ATPase activity, was calculated as difference between the K^+^-ATPase activities in the presence and absence of SCH28080.

### ^86^Rb^+^ transport assay

HEK293 cells on 6-well collagen-coated plates were incubated in 1 ml of solution composed of 144 mM NaCl, 0.5 mM MgCl_2_, 0.5 mM CaCl_2_, 1 mM RbCl (3×10^6^ cpm ^86^Rb^+^), 500 µM ouabain, 10 µM furosemide and 5 mM HEPES-NaOH (pH 7.4) for 10 min at 37°C. The ^86^Rb^+^ transport activity was determined as nanomoles of ^86^Rb^+^ transported/10^6^ cells in 1 min. Specific ^86^Rb^+^ transport activity was calculated as difference between the activity in the presence and absence of 50 µM SCH28080. After a 10-min incubation, the cells were washed and solubilized with 2 ml of the lysis buffer composed of 1% Nonidet P-40, 150 mM NaCl, 0.5 mM EDTA and 50 mM Tris-HCl (pH 7.4). Then, radioactivity of the sample was measured.

### Statistics

Results are presented as means ± s.e.m. Differences between groups were analyzed by one-way ANOVA, and correction for multiple comparisons was made by using Tukey's multiple comparison test. Comparison between two groups was made by using Student's *t* test. Statistically significant differences were assumed at *P*<0.05.
